# Supplementary material for: eCOMET: an R package for evaluating metabolic diversity and enrichment from LC‐MS/MS data to test ecological hypotheses from individuals to ecosystems
Source: New Phytol. 2026 Sep 16;252(4):1675–87. doi: 10.1111/nph.71589 (PMC13669803; doi:10.1111/nph.71589)
Supplement: Supplementary file 1 [file NPH-252-1675-s001.pdf]

New Phytologist Supporting Information

Article title: eCOMET: An R package for evaluating metabolic diversity and enrichment from LC-MS/MS data to test ecological hypotheses from individuals to ecosystems

Authors: Choi, Min-Soo, Forrister, Dale L., Dury, Guillaume J., Kang, Kyo Bin, Sedio, Brian E., Joo, Youngsung

Article acceptance date: [28 August 2026]

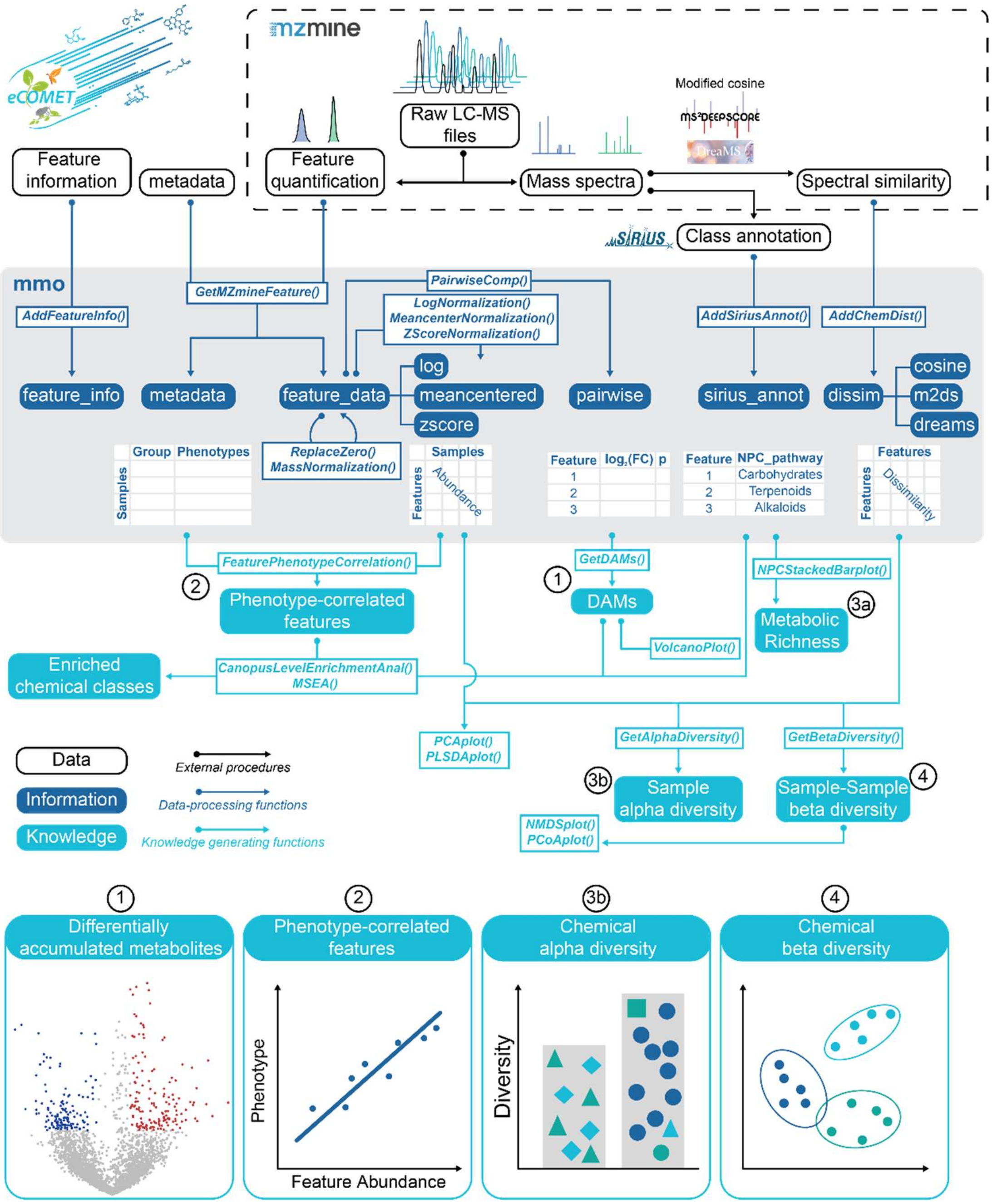

Supporting information Figure S1. Detailed structure of eCOMET from data integration to analytic modules.

Reference

Followings are citation for Supplementary Table S1(Sutter & Müller, 2011; Li *et al.*, 2020; Bakhtiari *et al.*, 2021; Defossez *et al.*, 2021; Endara *et al.*, 2022; Forrister *et al.*, 2022; Salgado *et al.*, 2023; Sun *et al.*, 2024; Bassi *et al.*, 2025; He *et al.*, 2025; MacNeill *et al.*, 2025; Tian *et al.*, 2025)

Bakhtiari M, Glauser G, Defossez E, Rasmann S. **2021**. Ecological convergence of secondary phytochemicals along elevational gradients. *New Phytologist* **229**: 1755–1767.

Bassi L, Hennecke J, Albracht C, Solbach MD, Rai A, Pinheiro Alves de Souza Y, Fox A, Zeng M, Döll S, Doan VC, *et al.* 2025. Plant species richness promotes the decoupling of leaf and root defence traits while species-specific responses in physical and chemical defences are rare. *New Phytologist* **246**: 729–746.

Defossez E, Pitteloud C, Descombes P, Glauser G, Allard PM, Walker TWN, Fernandez-Conradi P, Wolfender JL, Pellissier L, Rasmann S. **2021**. Spatial and evolutionary predictability of phytochemical diversity. *Proceedings of the National Academy of Sciences of the United States of America* **118**: 1–7.

Endara M-J, Soule AJ, Forrister DL, Dexter KG, Pennington RT, Nicholls JA, Loiseau O, Kursar TA, Coley PD. **2022**. The role of plant secondary metabolites in shaping regional and local plant community assembly. *Journal of Ecology* **110**: 34–45.

Forrister DL, Endara M, Soule AJ, Younkin GC, Mills AG, Lokvam J, Dexter KG, Pennington RT, Kidner CA, Nicholls JA, *et al.* 2022. Diversity and Divergence: Evolution of secondary metabolism in the tropical tree genus *Inga*. *New Phytologist*.

He Y, Junker RR, Xiao J, Lasky JR, Cao M, Asefa M, Swenson NG, Xu G, Yang J, Sedio BE. **2025**. Genetic and environmental drivers of intraspecific variation in foliar metabolites in a tropical tree community. *New Phytologist* **246**: 2551–2564.

Li D, Halitschke R, Baldwin IT, Gaquerel E. **2020**. Information theory tests critical predictions of plant defense theory for specialized metabolism. *Science Advances* **6**.

MacNeill FT, Hunter SG, Muth F, Sedio BE. **2025**. Nectar metabolomes contribute to pollination syndromes. *New Phytologist* **247**: 951–967.

Salgado AL, Glassmire AE, Sedio BE, Diaz R, Stout MJ, Čuda J, Pyšek P, Meyerson LA, Cronin JT. **2023**. Metabolomic Evenness Underlies Intraspecific Differences Among Lineages of a Wetland Grass. *Journal of Chemical Ecology* **49**: 437–450.

Sun L, He Y, Cao M, Wang X, Zhou X, Yang J, Swenson NG. **2024**. Tree phytochemical diversity and herbivory are higher in the tropics. *Nature Ecology and Evolution*.

Sutter R, Müller C. **2011**. Mining for treatment-specific and general changes in target compounds and metabolic fingerprints in response to herbivory and phytohormones in Plantago lanceolata. *New Phytologist* **191**: 1069–1082.

Tian M, Sun Y, Zhang G, Xu Y, Zhu J, Huang W, Wang Y, Zhang B, Li Z, Lin S, *et al.* 2025. Metabolomics navigates natural variation in pathogen-induced secondary metabolism across soybean cultivar populations. *Proceedings of the National Academy of Sciences* **122**: e2505532122.

.
